# Investigation Into the Effectiveness of Community Water Fluoridation and Dental Caries Experience in 4‐Year‐Old Aotearoa New Zealand Children: A National Repeated Cross‐Sectional Study From 2010 to 2022

**DOI:** 10.1111/cdoe.70080

**Published:** 2026-06-11

**Authors:** Tim Chambers, Philip J. Schluter, Frank Dean, Matt Hobbs, Jonathan Broadbent

**Affiliations:** ^1^ Ngāi Tahu Research Centre University of Canterbury Christchurch New Zealand; ^2^ Te Kaupeka Oranga, Faculty of Health, Te Whare Wānanga o Waitaha University of Canterbury Christchurch New Zealand; ^3^ School of Clinical Medicine, Primary Care Clinical Unit The University of Queensland Brisbane Australia; ^4^ Department of Oral Sciences University of Otago Dunedin Otago New Zealand

**Keywords:** community water fluoridation, dental caries, drinking water, Oral epidemiology, Oral health inequities

## Abstract

**Objectives:**

To provide contemporary national evidence on the effectiveness of community water fluoridation (CWF) for the prevention of early childhood caries (ECC) among 4‐year‐old children in Aotearoa New Zealand (NZ), and to examine potential effect modification by ethnicity and other sociodemographic characteristics.

**Methods:**

A national repeated cross‐sectional study was conducted using routinely collected B4 School Check data (2010–2022). Children aged 4 years were linked to a national spatial dataset of water distribution zones and council‐reported fluoridation histories to classify exposure over the assessment year and preceding 3 years. ECC was identified via the ‘lift‐the‐lip’ screening. Multilevel mixed‐effects modified Poisson regression models, with children nested within meshblocks, estimated incidence rate ratios (IRRs), adjusting for ethnicity, area‐level deprivation, age, sex, rurality, and assessment year. Two‐factor interactions were examined.

**Results:**

Among 630 926 children, 14.2% had ECC. In crude analyses, ECC appeared more common in fluoridated areas; however, after adjustment for sociodemographic characteristics, CWF was associated with a lower rate of ECC (IRR = 1.13; 95% CI: 1.11–1.15 for non‐fluoridated vs. fluoridated). A significant interaction between CWF and ethnicity was observed. The protective association was strongest among European/Other children (IRR = 1.25; 95% CI: 1.21–1.29) and attenuated among Māori and Asian children, with minimal effect among Pacific children. The ethnicity‐weighted population attributable fraction was 4.4%, equating to approximately 282 ECC cases annually.

**Conclusion:**

Findings are consistent with CWF being an effective population‐level oral health intervention. However, its association with caries experience varies by ethnicity, highlighting the importance of addressing structural inequities alongside universal preventive strategies.

## 1 Introduction

In 2024, 42% of 5‐year‐old children in Aotearoa New Zealand (NZ) had evidence of dental caries, making it the most prevalent chronic health condition in children [1]. The rate of child hospitalisations requiring a general anaesthetic for dental treatment has been estimated to be 10.3 per 1000 for 0‐4y, 13.7 per 1000 for 5‐9y and 2.0 per 1000 for 10‐14y from 2010–2019 [2].

Community water fluoridation (CWF) has been used for decades in many countries, including NZ. NZ has recently expanded national coverage of CWF from 50.7% to 62.4% of the population, through the implementation of the Health (Fluoridation of Drinking Water) Amendment Act 2021 [3]. On July 27th 2022, 14 Territorial Authorities (TAs) were instructed to prepare to fluoridate water in one or more water distribution zones (WDZ) [4]. These directives have significantly reduced inequities in access to CWF [5]. However, legal [6] or political [7] challenges may lead to the CWF expansion being halted or reversed.

Unfortunately, evidence on the effectiveness of CWF for dental caries prevention is outdated. A 2015 Cochrane review included only two studies published post‐1975 and concluded that contemporary evidence on the effectiveness of CWF for the prevention of dental caries is required [8]. Partly addressing this limitation, a number of additional large observational studies have reinforced the effectiveness of CWF [9, 10, 11]. For example, in 2018, one large cohort study of 24 000 Australian children found that children with lower lifetime CWF exposure had rates of tooth decay approximately two times greater than children living in a CWF area all their life [11]. However, additional contemporary evidence is required to update estimates of the effectiveness of CWF due to the greater focus on preventive dental care and widespread coverage of fluoride toothpastes.

Previous NZ evidence on CWF effectiveness is persuasive but has limitations [12, 13, 14, 15, 16]. During the past 20 years, three cross‐sectional studies reported that children living in fluoridated areas have from 17% to 40% lower rates of dental caries than children living in non‐fluoridated areas once adjusted for age, sex, ethnicity and area‐level deprivation [12, 14, 15]. One of these studies used individual‐level data from the B4 School Check (B4SC), a national screening programme of 4‐year‐old children. That study used data from 2010 to 2016, used a rudimentary method of determining fluoridation status for children, employed statistical techniques that can over‐estimate risk [17], and did not explore the interaction effects of key sociodemographic characteristics such as ethnicity. Taken together, these limitations may have led to an underestimation of the true benefit of CWF, both through misclassification of children's fluoridation exposure and by not fully accounting for structural factors related to ethnicity that may modify its effect.

Due to these knowledge gaps, and the opportunity afforded by newly‐available data, this study integrates individual‐level data from 2010 to 2022 from the B4SC, a national dataset on the spatial extent of water distribution zones (WDZ) of publicly‐owned water supplies [18] and data collected from councils on the start and finish dates of their CWF schemes (as well as any interruptions greater than 6 months) to investigate the association between CWF for dental caries prevention in children aged 4 years. Potential interaction effects were examined by sociodemographic characteristics to examine potential effect modification by ethnicity, sex, neighbourhood deprivation, and age.

## 2 Methods

### 2.1 Study Design

This was a national repeated cross‐sectional study. NZ's Health and Disability Ethics Committee defined this study as minimal‐risk observational research not needing formal ethics committee review or further consents for use of data. Use of the B4SC data was approved by the NZ Ministry of Health (MoH).

### 2.2 Population Studied

This research used data for NZ children aged 4 years who had their B4SC assessment from July 2010 to June 2022. To be eligible for a B4SC, the age of assessment is meant to be between 4 years and 5 years 7 days, so there is some variation in age of assessment.

### 2.3 Outcome Measurement

Our primary outcome was dental caries as identified in the “lift the lip” oral health screen conducted as part of the B4SC [19]. This screening is conducted by trained nurses in accordance with the Ministry of Health's standardised protocol [19]. Nurses receive training in the lift the lip assessment, including the use of clinical guidance and photographic reference examples. Children's teeth are classified on a scale from one to six, where one represents no visible caries, while any score two or higher meets the criteria for early childhood caries (ECC) at age 4 years [19], and the child is referred for treatment (approximately 15% of NZ 4‐year‐olds) [14]. Note, this is substantially lower than the prevalence reported from NZ's Community Oral Health Service at age 5 years (43% in 2024), suggesting it is likely that considerable early or uncavitated caries remains undetected in the age 4 B4SC [1].

### 2.4 Exposure Assessment

The key assumption in this repeated cross‐sectional study design is that fluoride exposure at age four (B4SC assessment) is representative of lifetime exposure to that point. Data were obtained from all TAs about the start and end dates for CWF and any interruptions to CWF greater than 6 months. Any year with an interruption of greater than 6 months was treated as non‐fluoridated.

Three categories of fluoridation status were created: (1) entirely fluoridated (where the assessment year and previous 3 years were fluoridated); (2) entirely non‐fluoridated (where the assessment year and previous 3 years were not fluoridated); and (3) mixed fluoridation (where at least 1 year in four (assessment year plus three previous years) was discordant (either fluoridated or non‐fluoridated)). In the main analysis, mixed fluoridation was recoded as “entirely fluoridated”, while in a sensitivity analysis, all mixed were included as entirely non‐fluoridated as well as excluding mixed supplies.

For each year from 2010 to 2022, a fluoridation status variable was generated for each WDZ (based on data from 2007 to 2022). The development of a national spatial dataset of all publicly owned WDZ in NZ has recently been documented [18]. This dataset covers ~88% of the total population, with the remaining population drawing water from their own private supplies [18] In validation analyses, this dataset has demonstrated a high positive predictive value (accurately identifying an individual as being on a public water supply) [20].

Each child was linked to a WDZ based on their meshblock of residence taken as part of the B4SC assessment and used this as a proxy for exposure from age 0 to the year of assessment. A meshblock is the smallest geographic unit used by Statistics New Zealand, which usually includes between 30–60 dwellings and is no larger than 120 dwellings (representative of a city block).

### 2.5 Sociodemographic Variables

Sociodemographic variables derived from the B4SC data set included sex, age, and ethnicity. Sex was categorised as female and male. Age (in months) was calculated from B4SC assessment and birth dates. Ethnicity was based on MoH protocols where children with multiple identifications were assigned a single ethnicity via the prioritisation hierarchy: (1) Māori; (2) Pacific; (3) Asian; (4) Middle Eastern, Latin American, and African; and (5) European/other.

A meshblock defined level of neighbourhood deprivation was measured using the New Zealand Deprivation Index 2013 (NZDep2013) [21] and NZDep2018 [22] linked based on the child's recorded residential meshblock at their B4SC assessment via the 2013 and 2018 censuses. The NZDep combines census data relating to income, home ownership, employment, qualifications, family structure, housing, access to transport, and communications into a single measure [21, 22]. Each meshblock is assigned a score from 1 (least deprived) to 10 (most deprived) based on decile splits, which were aggregated into quintiles for our analysis.

Rural/urban classification was determined by linking a child's residential meshblock to the New Zealand Urban Rural Standard Classification: (1) main urban (≥ 100 000 people); (2) large urban (30 000–99 999 people); (3) medium urban (10 000–29 999 people); (4) small urban (1000–9999 people); (5) rural (200–1000 people); and (6) other rural (< 200 people). Any assessment between 2010 to 2017 was linked to 2013 NZDep or Urban/Rural classifications, while assessments from 2018 onwards were linked to the 2018 classification.

### 2.6 Statistical Analysis

The Reporting of studies Conducted using Observational Routinely‐collected Data (RECORD) guidelines were used to inform reporting of this study [23]. Comparisons between the exposure groups and the sociodemographic characteristics were made using Pearson's χ^2^ test. For the primary analyses, fluoridation exposure was dichotomised to include entirely fluoridated vs. entirely non‐fluoridated groups, where WDZ classified as mixed fluoridation were recoded to entirely fluoridated. A secondary sensitivity analysis followed, with exposure dichotomised as entirely fluoridated vs. entirely non‐fluoridated groups, where mixed fluoridated WDZ were recoded as entirely non‐fluoridated. Because ECC is not a rare outcome, and logistic regression models are known to provide biased estimates of relative risk in such instances, modified Poisson regression models were instead employed [17]. Further, as children were nested within meshblocks, multilevel mixed‐effects models were employed with children nested within meshblocks. In these models, meshblocks were treated as random intercepts. Mirroring procedures employed elsewhere [14], sociodemographic and potentially confounding variables were initially considered and sequentially added to develop a main effects model. Next, all 2‐factor interaction terms, including those with fluoridation status, were considered and sequentially added to the main effects model until there was no improvement in the Bayesian Information Criterion (BIC) [24]. In the regression models, age was centred at its median (51 months). Reference categories were: female (sex), European/Other (ethnicity), NZDep quintile 1 (least deprived), urban—major (rural/urban classification), and mid‐study year (2016) for assessment year. All analyses were performed using Stata SE, version 18.0 (StataCorp LLC, College Station, Texas, USA).

## 3 Results

### 3.1 Study Sample

The raw B4SC file contained assessment data for 732 622 children from 2010 to 2022. Figure 1 shows the inclusion flow chart and exclusion criteria for the study cohort. Children were excluded from the cohort due to not having a valid oral health assessment (*n* = 77 236, 10.5%); missing address information (*n* = 22 350, 3.1%); and missing other covariate information (*n* = 2110, 0.3%). The final analytic sample included 630 926 4‐year‐old children (86.1% of initial sample).

**FIGURE 1:**
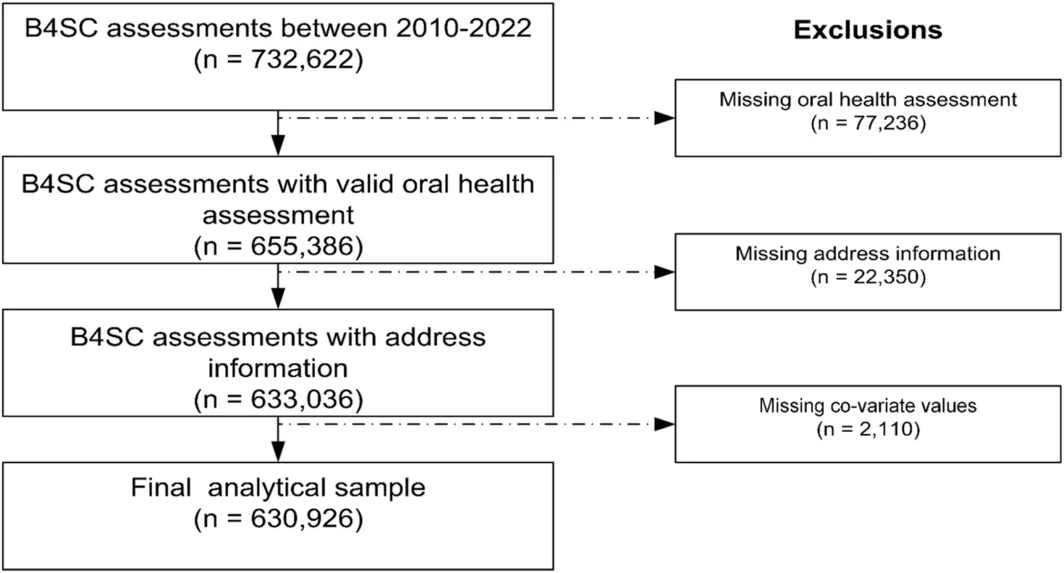
Study population flow diagram with study exclusions.

Overall, included children had a median age of 51 months (4.25 years) at their B4SC assessment and 307 481 (48.7%) were female (Table 1). European/Other was the primary ethnic identity for 50.7% of the sample, 25.9% lived in the most deprived areas, and 84.9% resided within an urban area.

**TABLE 1:** Sociodemographic characteristics of the eligible sample (*n* = 630 926) overall and partitioned by the fluoridation exposure groups: Fluoridated (*n* = 317 371), mixed fluoridation (*n* = 24 785), and unfluoridated (*n* = 288 770).

Characteristic	Overall		Fluoridated		Mixed		Unfluoridated	
	*n*	(%)	*n*	(%)	*n*	(%)	*n*	(%)
Sex								
Female	307 481	(48.7)	154 538	(48.7)	12 117	(48.9)	140 826	(48.8)
Male	323 445	(51.3)	162 833	(51.3)	12 668	(51.1)	147 944	(51.2)
Ethnicity								
Māori	144 908	(23.0)	61 957	(19.5)	7876	(31.8)	75 075	(26.0)
Pacific	62 440	(9.9)	50 500	(15.9)	1642	(6.6)	10 298	(3.6)
Asian	92 394	(14.6)	68 519	(21.6)	2574	(10.4)	21 301	(7.4)
MELAA	11 553	(1.8)	7874	(2.5)	491	(2.0)	3188	(1.1)
European/Other	319 631	(50.7)	128 521	(40.5)	12 202	(49.2)	178 908	(62.0)
Area‐level deprivation								
Q1 (least deprived)	109 341	(17.3)	53 872	(17.0)	3468	(14.0)	52 001	(18.0)
Q2	112 365	(17.8)	56 319	(17.7)	3037	(12.3)	53 009	(18.4)
Q3	118 251	(18.7)	54 291	(17.1)	4016	(16.2)	59 944	(20.8)
Q4	127 680	(20.2)	60 979	(19.2)	5421	(21.9)	61 280	(21.2)
Q5 (most deprived)	163 289	(25.9)	91 910	(29.0)	8843	(35.7)	62 536	(21.7)
Rural/urban classification								
Urban – major	323 077	(51.2)	241 025	(75.9)	12 986	(52.4)	69 066	(23.9)
Urban – large	98 817	(15.7)	41 443	(13.1)	5657	(22.8)	51 717	(17.9)
Urban – medium	51 695	(8.2)	18 940	(6.0)	1381	(5.6)	31 374	(10.9)
Urban – small	62 187	(9.9)	9226	(2.9)	2979	(12.0)	49 982	(17.3)
Rural centre	16 050	(2.5)	1140	(0.4)	314	(1.3)	14 596	(5.1)
Rural or remote	79 100	(12.5)	5597	(1.8)	1468	(5.9)	72 035	(24.9)

Abbreviation: Middle Eastern, Latin American, and African. Q1: Quintile 1.

### 3.2 Fluoridation Exposure

In total, 628 water distribution zones (WDZ) were included in the nationwide spatial dataset. Of these, 483 (76.9%) WDZ were never fluoridated at any point in time (Table S1), while a further 8 (1.3%) WDZ had been fluoridated in the past but had ceased at some point prior to 2007 during the observation period used in this analysis. An additional 106 (16.9%) WDZ were fluoridated for the whole study period. Finally, 31 (4.9%) WDZ had a mixed fluoridation status.

Across these WDZ's 288 770 (45.8%) children were classified as living in unfluoridated areas, 317 371 (50.3%) in fluoridated areas, and 24 785 (3.9%) in areas of mixed fluoridation status. A greater proportion of children classified within the mixed or unfluoridated exposure groupings were Māori children or living in small urban, rural, or remoted regions, whereas a greater proportion of children classified in the fluoridated or mixed exposure groups lived in the most deprived regions of NZ (Table 1).

### 3.3 Oral Health

In total, 89 591 (14.2%) of children experienced ECC. Table 2 gives the distribution of ECC status across sociodemographic characteristics overall and partitioned by the fluoridation exposure groups. Clear differential patterns can be identified, with greater rates of ECC associated with higher area‐level deprivation, and with Pacific and Māori children experiencing higher rates than European/Other children.

**TABLE 2:** Distribution of caries status across sociodemographic characteristics of the eligible sample (*n* = 630 926) overall and partitioned by the fluoridation exposure groups: Fluoridated (*n* = 317 371), mixed fluoridation (*n* = 24 785), and unfluoridated (*n* = 288 770).

Characteristic	Overall		Fluoridated		Mixed		Unfluoridated	
	*n*	(%)	*n*	(%)	*n*	(%)	*n*	(%)
Sex								
Female	41 836	(13.6)	22 843	(14.8)	1465	(12.1)	17 528	(12.4)
Male	47 755	(14.8)	25 861	(15.9)	1715	(13.5)	20 179	(13.6)
Ethnicity								
Māori	32 648	(22.5)	13 244	(21.4)	1656	(21.0)	17 748	(23.6)
Pacific	18 774	(30.1)	15 533	(30.8)	404	(24.6)	2837	(27.5)
Asian	15 735	(17.0)	11 481	(16.8)	350	(13.6)	3904	(18.3)
MELAA	1609	(13.9)	1156	(14.7)	65	(13.2)	388	(12.2)
European/Other	20 825	(6.5)	7290	(5.7)	705	(5.8)	12 830	(7.2)
Area‐level deprivation								
Q1 (least deprived)	7341	(6.7)	3850	(7.1)	181	(5.2)	3310	(6.4)
Q2	9684	(8.6)	5224	(9.3)	205	(6.8)	4255	(8.0)
Q3	13 026	(11.0)	6284	(11.6)	379	(9.4)	6363	(10.6)
Q4	18 442	(14.4)	9308	(15.3)	607	(11.2)	8527	(13.9)
Q5 (most deprived)	41 098	(25.2)	24 038	(26.2)	1808	(20.4)	15 252	(24.4)
Rural/urban classification								
Urban – major	48 749	(15.1)	38 742	(16.1)	1580	(12.2)	8427	(12.2)
Urban – large	15 745	(15.9)	5785	(14.0)	828	(14.6)	9132	(17.7)
Urban – medium	5455	(10.6)	2316	(12.2)	100	(7.2)	3039	(9.7)
Urban – small	9400	(15.1)	1259	(13.6)	524	(17.6)	7617	(15.2)
Rural centre	2257	(14.1)	153	(13.4)	53	(16.9)	2051	(14.1)
Rural or remote	7985	(10.1)	449	(8.0)	95	(6.5)	7441	(10.3)

Abbreviation: Middle Eastern, Latin American, and African. Q1: Quintile 1.

### 3.4 Examining the Association Between CWF and Oral Health

Crude analysis showed that 48 704 (15.3%) children residing in fluoridated areas had ECC higher than the 37 707 (13.1%) and 3180 (12.8%) seen in the unfluoridated and mixed fluoridated areas, respectively. A crude multi‐level mixed effect analysis comparing fluoridated/mixed group against the unfluoridated group yielded an incidence rate ratio (IRR) of 0.92 (95% confidence interval [CI]: 0.91, 0.94) which suggests that living in unfluoridated areas was associated with less caries experience. However, variations in ECC patterns across sociodemographic characteristics between the fluoridation exposure groups are also evident from Table 2. The crude analysis ignores these differing fluoridation exposure and caries experience patterns, behoving statistical adjustment.

### 3.5 Adjusted Analyses

Initially, a main effects model was derived. Forward selected variables minimising the BIC statistic consisted of ethnicity (BIC = 495368.4), followed by area‐level deprivation (BIC = 490650.8), age (BIC = 488908.7), B4SC assessment year (BIC = 488606.9), sex (BIC = 488488.2), and rural/urban classification (BIC = 488456.1). A quadratic term for age was also considered, but resulted in a worsening of the BIC statistic, and was thus not included (BIC = 488458.7). All included variables were associated with caries experience in this main effects model (all *p* < 0.001).

Next, all 2‐factor interactions were considered. Interactions minimising the BIC statistic included ethnicity × NZDep (BIC = 488110.9), followed by fluoridation exposure × ethnicity (BIC = 488081.7), and ethnicity × age (BIC = 488070.3). No other 2‐factor interactions yielded a lower BIC statistic, and thus model building terminated. The included interaction terms were also significantly associated with caries experience (all *p* < 0.001). While all identified interaction terms were retained in the final model, only the fluoridation exposure × ethnicity interaction is presented (Figure 2), as this was the primary interaction of interest.

**FIGURE 2:**
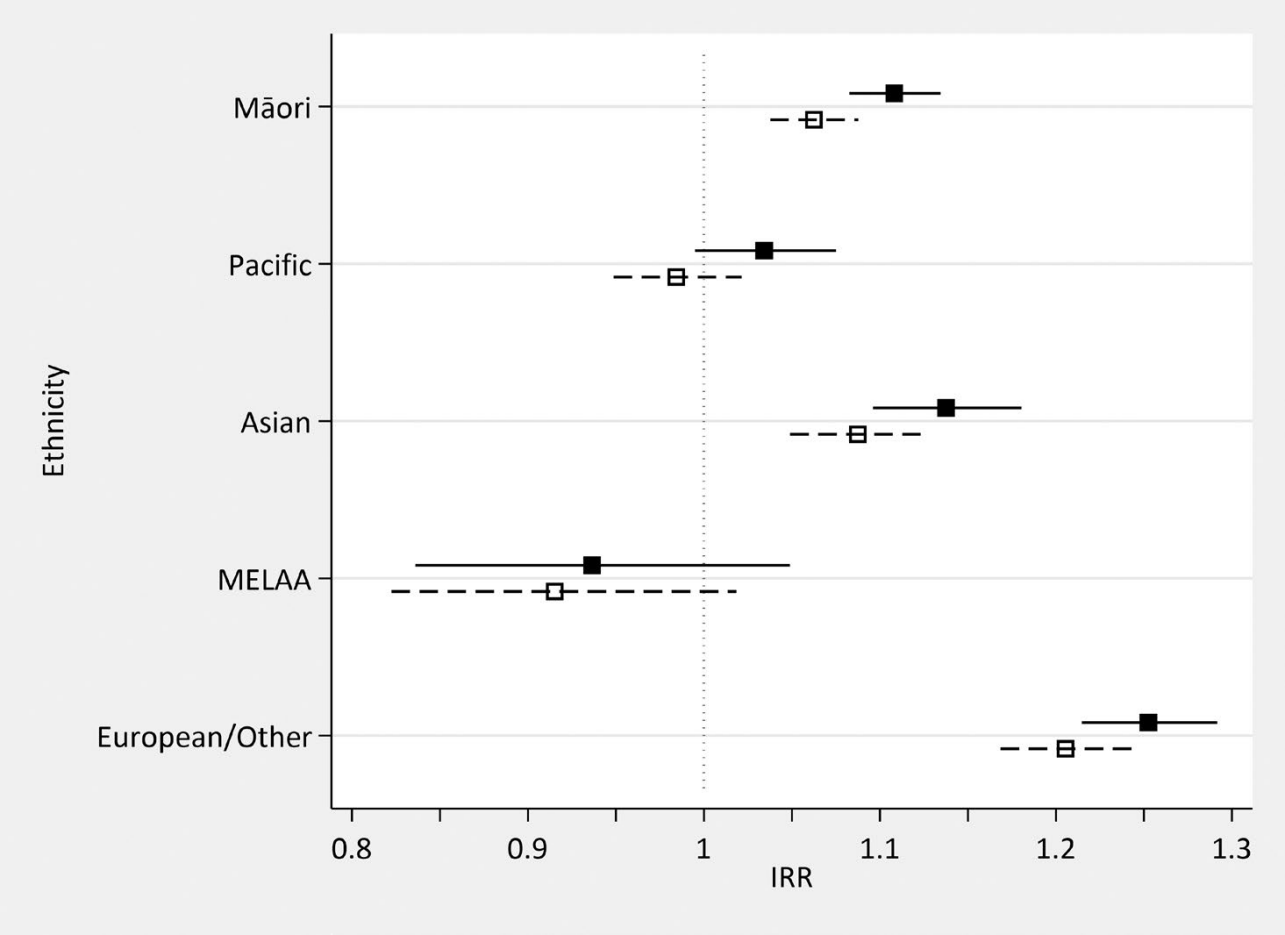
Fluoridation IRR effect size estimates and associated 95% CI from adjusted multilevel mixed‐effects modified Poisson regression models (*n* = 630 926). The solid boxes and lines represent fluoridation exposure characterised by fluoridated/mixed vs. unfluoridated groups whereas the hollow boxes and dashed lines represent fluoridation exposure characterised by fluoridated vs. mixed/unfluoridated groups. Estimates are presented for the fluoridation exposure × ethnicity interaction, with all other covariates held at their reference values.

Figure 2 presents the IRR effect size estimates and associated 95% CI from the final adjusted multilevel mixed‐effects modified Poisson regression models (figures used to populate Figure 2 available in Table S2). Due to the significant fluoridation exposure × ethnicity interaction, this implies that the association between CWF and oral health outcomes differed by ethnicity.

For the primary exposure, characterising mixed supplies as entirely fluoridated, the largest estimated effect size was European/Other children (IRR = 1.25; 95% CI: 1.21, 1.29), followed by Asian (IRR = 1.14; 95% CI: 1.10, 1.18) and Māori (IRR = 1.11; 95% CI: 1.08, 1.13) children. However, the impact of fluoridation appeared negligible for Pacific (IRR = 1.03; 95% CI: 1.00, 1.07) and MELAA (IRR = 0.94; 95% CI: 0.84, 1.05) children, once differences in measured sociodemographic variables were accounted for. In the second model, where mixed supplies were treated as entirely nonfluoridated, the same patterns emerged—except the estimated IRR were generally dampened (e.g., European/Other children IRR = 1.21; 95% CI: 1.17, 1.24).

### 3.6 Population Attributable Fraction

In total, 89 591 children had a positive ‘lift‐the‐lip’ B4SC screening over the study period. The ethnicity‐weighted population‐attributable fraction was 4.4% (95% CI 3.7–5.2) suggesting 3942 (95% CI 3315–4659) cases were attributable to non‐fluoridation of community water supplies, or 282 cases of ECC detected by ‘lift‐the‐lip’ in the B4SC screening at age 4 years annually.

## 4 Discussion

These findings provide contemporary evidence of an association between CWF and lower caries experience at age 4 years using nationwide individual‐level data. However, the findings also highlight the complex interactions between CWF, ethnicity, area‐level deprivation, and oral health. These interactions highlight the limitations of ecological analyses and provide evidence of the impact of confounding by sociodemographic characteristics.

The crude IRR for unfluoridated areas suggested CWF was associated with greater ECC risk (IRR 0.91, 95% CI 0.90, 0.94). This is consistent with some other previous unadjusted analyses, particularly those using the Ministry of Health oral health data at age 5 or 12—see Supplementary Figure S1. Unfortunately, these types of analyses have been frequently cited in policy and legal proceedings to advocate for removing CWF (for instance, see the reference to evidence put forward in *New Health New Zealand Inc v South Taranaki District Council* [2017] 2 NZLR 13 at [145]). However, in our adjusted model, the IRR is reserved and shows a protective effect of CWF (IRR 1.13, 95% CI 1.11–1.15). This is explained by a substantially higher proportion of the children living in the highest deprivation and children of non‐European ethnicity living in fluoridated areas (29% and 60%, respectively) than non‐fluoridated areas (22% and 38%, respectively)—who are also systematically more likely to have higher rates of ECC.

The main adjusted effect of CWF suggests a 12% lower rate of advanced ECC in children aged 4, which is broadly consistent with previous studies [12, 14, 15] In particular, it is similar, albeit lower than, the 21% observed in a previous analysis using a smaller number of observations, different exposure assessment and statistical methods [14] Other NZ studies have reported reductions in caries experience ranging from approximately 17% to 40% [12, 14, 15], although these estimates are not directly comparable to this current study due to methodological differences in study design, exposure assessment, and outcome measures. For example, Ministry of Health reporting has shown substantially higher mean dmft scores in non‐fluoridated areas, corresponding to an absolute mean difference of approximately one dmft. These methodological differences likely contribute to variation in reported effect sizes, but overall, the findings remain consistent in direction, supporting a protective association between CWF and dental caries.

The findings demonstrated persistent and substantial inequities in oral health by ethnicity. In adjusted models, Māori, Pacific, Asian, and MELAA children had rates of ECC that were 2.6‐, 3.4‐, 2.6‐, and 2.0‐fold higher, respectively, than those of NZE children. These findings are consistent with previous findings of greater caries and dental‐related hospitalisation for Māori than non‐Māori.

While the main effect of CWF was protective overall, age‐centred analyses suggested variation in effectiveness by ethnicity. At both 51 and 60 months, the protective effect of CWF was greater for NZE children than for all other ethnic groups. Biases related to the cross‐sectional study design and exposure assessment could partly explain these differences. Firstly, previous NZ evidence has demonstrated there is greater residential mobility among children of Māori and Pacific ethnicity [25] As a result, children of Māori and Pacific ethnicity are more likely to experience misclassification of CWF exposure at the time of B4SC assessment compared with NZE children. This would be expected to result in non‐differential exposure misclassification, biasing estimates toward the null and potentially underestimating the protective effect of CWF in these groups, as discussed further in the limitations section. Another possible explanation is differential fluoridation performance in water supplies serving Māori communities. A national analysis of CWF monitoring data found that only 56.5% of person‐years of exposure for Māori were within the optimal fluoride range (0.7–1.0 ppm), compared with 61.4% for non‐Māori, suggesting systemic inequities in both exposure and benefit [5] Lastly, as a population‐level intervention, CWF is expected to shift the distribution of caries severity leftwards rather than eliminate disease entirely. Given that Māori and Pacific children are over‐represented in the right tail of the severity distribution (e.g., hospitalisations), this shift is likely to reduce disease severity and avert the most severe outcomes, even where caries persist. Among European children, who tend to have lower baseline severity, the same shift may result in complete prevention of caries for a subset of the population.

### 4.1 Strengths and Limitations

A major strength of this study is its near whole‐of‐population coverage, drawing on 13 years of data from a national oral health surveillance programme. The analysis provides additional contemporary evidence on the effectiveness of CWF while controlling for key confounders. The large sample size also enabled investigation of potential interaction effects between CWF and important covariates. The availability of individual‐level data also mitigates possible ecological fallacy bias, an issue highlighted by analyses of oral health data and CWF status presented above. The study also used an updated exposure assessment that leveraged national dataset of the spatial extent of WDZ, dates for start and finish of and interruptions to CWF and considered fluoridation status in years prior to the B4SC assessment date.

The primary limitation of this study is its cross‐sectional design. The analysis assumes that CWF exposure remains constant from birth to age four, meaning reverse causation cannot be excluded. In a prospective longitudinal cohort study of children in NZ, 34% of the cohort did not move between birth and age four, while 26% had one move and 40% two or more relocations [26] However, only 9% of all relocations in the same cohort at age two were over 50 km, suggesting most children likely retain their CWF exposure as fluoridation status is represented at the city‐scale [27] Nevertheless, this mobility introduces potential misclassification bias, likely attenuating effect estimates toward the null, as children in the entirely non‐fluoridated group may have received the intervention in part (e.g., CWF from age 0 to 3) and children in the entirely fluoridated group may not have received the intervention until age 4 (e.g., non‐fluoridated until age 3). As stated earlier in the discussion, this bias is likely to have affected children of Māori and Pacific ethnicity the most.

Another limitation is the use of fluoridation status as a proxy for fluoride exposure. This approach assumes water supplies are consistently fluoridated at optimal concentrations (0.7–1.0 ppm). While this assumption holds in countries with high compliance, in NZ, water is underdosed approximately 40% of the time, with variation by ethnicity and area‐level deprivation [5].

## 5 Conclusions

This study reinforces CWF as an effective population level oral health intervention. However, the association between CWF and lower dental caries experience differed substantially by children's ethnicity. Further research is warranted to determine plausible explanations for this differential effectiveness.

## Funding

The project was funded by the Ministry of Health Oral Health Fund and the Clare Foundation. The funders did not have any input into the study design, collection, analysis and interpretation of data, writing of the report, and decision to submit the article for publication.

## Ethics Statement

NZ's Health and Disability Ethics Committee defined this study as minimal‐risk observational research not needing formal ethics committee review or further consents for use of data This study used routinely collected, de‐identified surveillance data obtained from the B4 School Check maintained by the Ministry of Health. Use of the B4SC data was approved by the NZ Ministry of Health (MoH) As the study involved secondary analysis of routinely collected and fully de‐identified data, informed consent from individual participants was not required and was formally waived by NZ's Health and Disability Ethics Committee.

## Conflicts of Interest

The authors declare no conflicts of interest.

## Supporting information


**Table S1:** Initial fluoridation status defined at the WDZ‐level across the study period.
**Table S2:** Fluoridation IRR effect size estimates and associated 95% CI from adjusted multilevel mixed‐effects modified Poisson regression models (*n* = 630 926).
**Figure S1:** Percentage of children aged 5 with dental caries as reported by Ministry of Health by fluoridation status.

## Data Availability

The data that support the findings of this study are available from Ministry of Health. Restrictions apply to the availability of these data, which were used under license for this study. Data are available from the author(s) with the permission of Ministry of Health.
