# Supplementary material for: Investigation Into the Effectiveness of Community Water Fluoridation and Dental Caries Experience in 4‐Year‐Old Aotearoa New Zealand Children: A National Repeated Cross‐Sectional Study From 2010 to 2022
Source: Community Dent Oral Epidemiol. 2026 Jun 11;54(5):622–30. doi: 10.1111/cdoe.70080 (PMC13669797; doi:10.1111/cdoe.70080)
Supplement: Supplementary file 1 [file CDOE-54-622-s001.docx]

# Supplementary material

In total, there are 628 water distribution zones (WDZ) in the nationwide spatial dataset. Of these, 483 (76.9%) WDZ that have never been fluoridated at any point in time. A further 8 (1.3%) WDZ have never been fluoridated in the observation period used in this analysis e.g. fluoridation stopped before 2007. An additional 106 (16.9%) WDZ were fluoridated for the whole period. Then there were 31 (4.9%) WDZ that had a mixed fluoridation status. The mixed fluoridation WDZ were broken down into 15 (2.4%) WDZ which had started fluoridation after 2006 (and therefore had at least 1 year where there was mixed fluoridation) and 16 (2.5%) WDZ which had finished fluoridation prior to 2022.

## **Supplementary Table 1**. Initial fluoridation status defined at the WDZ-level across the study period.

| **Fluoridation status** | | **WDZ** | | **WDZ-years** |
| --- | --- | --- | --- | --- |
|  |  | n | % |  |
| **Total** | Total | 628 | 100 | 8,164 |
| **Entirely non-fluoridated** | At any point | 483 | 76.9 | 6,279 |
|  | In study period | 8 | 1.3 | 104 |
| **Entirely fluoridated** | In study period | 106 | 16.9 | 1,378 |
| **Mixed fluoridation** | Started 2007 or after | 15 | 2.4 | 195 |
|  | Finished prior to 2022^b^ | 16 | 2.5 | 208 |

^a^ e.g. Started in 2007 so first real year of complete fluoridation would be 2011.
^b^ e.g. Finished in 2016 so all following years are incomplete fluoridation

The simplest approach would have been to exclude all mixed fluoridation zones but to minimize the number of exclusions we included any year of observation where the assessment year and previous three years were either entirely fluoridated or non-fluoridated.

First, we resampled the dataset to create an observation year from 2010 to 2022 for all 628 WDZ zones, which created 8,164 WDZ years. In total, there were **403 WDZ** years that were affected by either a late start date or early end date for CWF (supplementary Figure 1). In total 160 (39.7%) WDZ years were recoded as being non-fluoridated and 80 (19.9%) WDZ years were coded as being fluoridated, leaving 163 (40.4%) WDZ years being coded as mixed fluoridation. In combination with the 70 WDZ years impacted by interruptions, there were a total of 233 WDZ years coded as mixed fluoridation (2.9% of the total WDZ years).

## **Supplementary Table 2**. Fluoridation IRR effect size estimates and associated 95% CI from adjusted multilevel mixed-effects modified Poisson regression models (n=630,926).

|  | **Main Analysis^1^** | | **Sensitivity 1^2^** | | **Sensitivity 2^3^** | |
| --- | --- | --- | --- | --- | --- | --- |
| **Ethnicity** | **IRR** | **(95% CI)** | **IRR** | **(95% CI)** | **IRR** | **(95% CI)** |
| Māori | 1.11 | (1.08, 1.13) | 1.06 | (1.04, 1.09) | 1.10 | (1.07, 1.13) |
| Pacific | 1.03 | (1.00, 1.07) | 0.98 | (0.95, 1.02) | 1.03 | (0.99, 1.07) |
| Asian | 1.14 | (1.10, 1.18) | 1.09 | (1.05, 1.13) | 1.13 | (1.09, 1.17) |
| MELAA^a^ | 0.94 | (0.84, 1.05) | 0.92 | (0.82, 1.02) | 0.93 | (0.83, 1.04) |
| European/Other | 1.25 | (1.21, 1.29) | 1.21 | (1.17, 1.24) | 1.25 | (1.20, 1.29) |

^1^ Entirely Fluoridated & mixed vs unfluoridated groups
^2^ Entirely Fluoridated vs unfluoridated groups & mixed
^3^ Entirely Fluoridated vs unfluoridated – mixed excluded.

## Supplementary Figure 1. Percentage of children aged 5 with dental caries as reported by Ministry of Health by fluoridation status.
